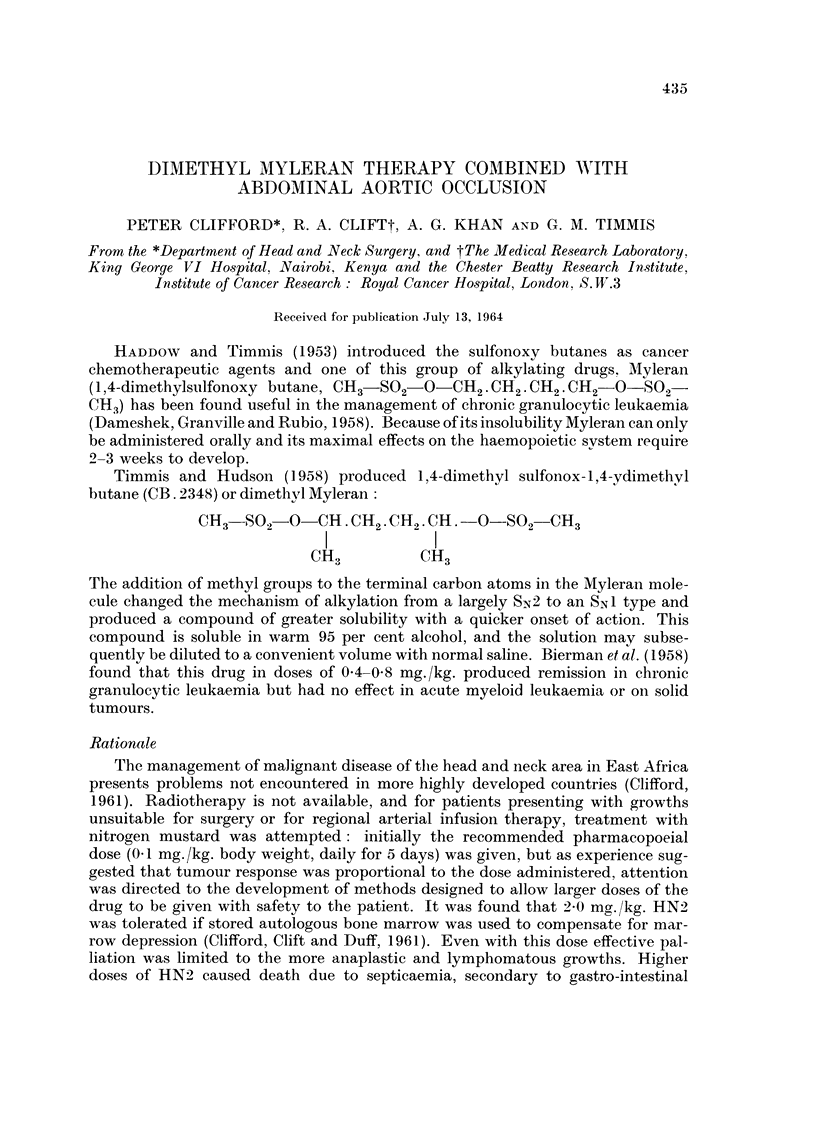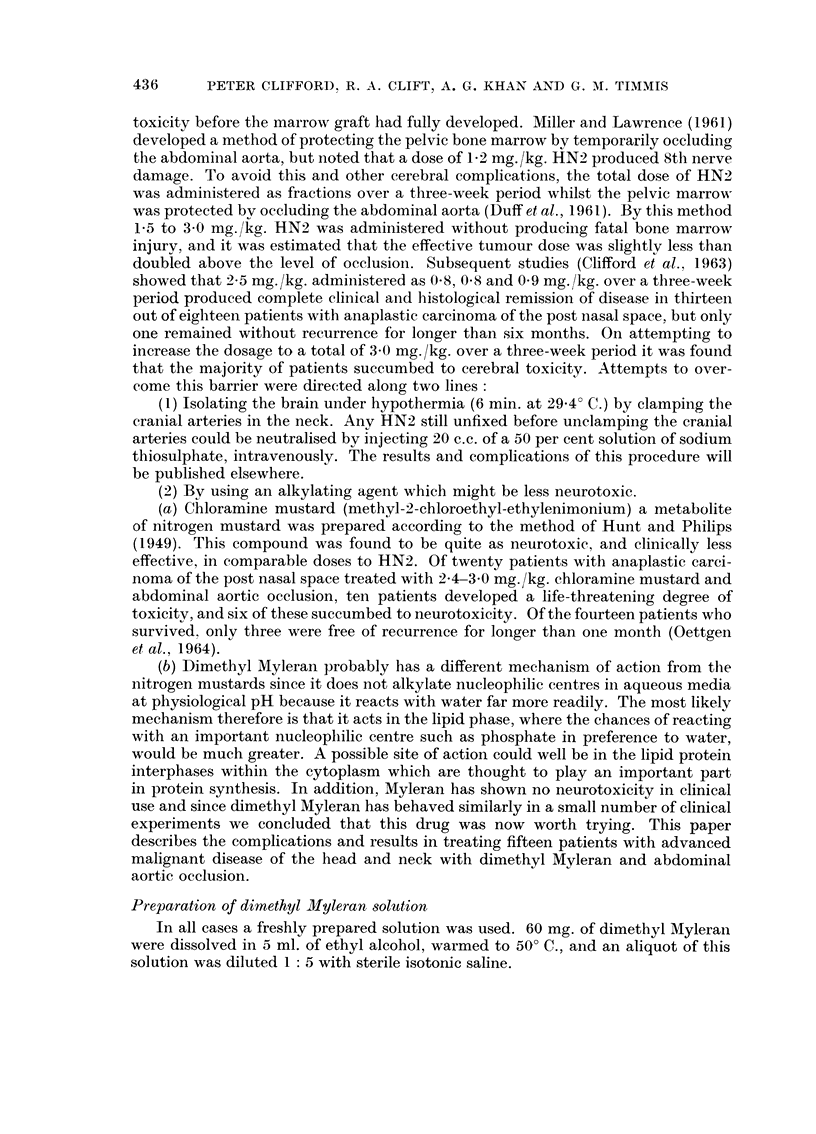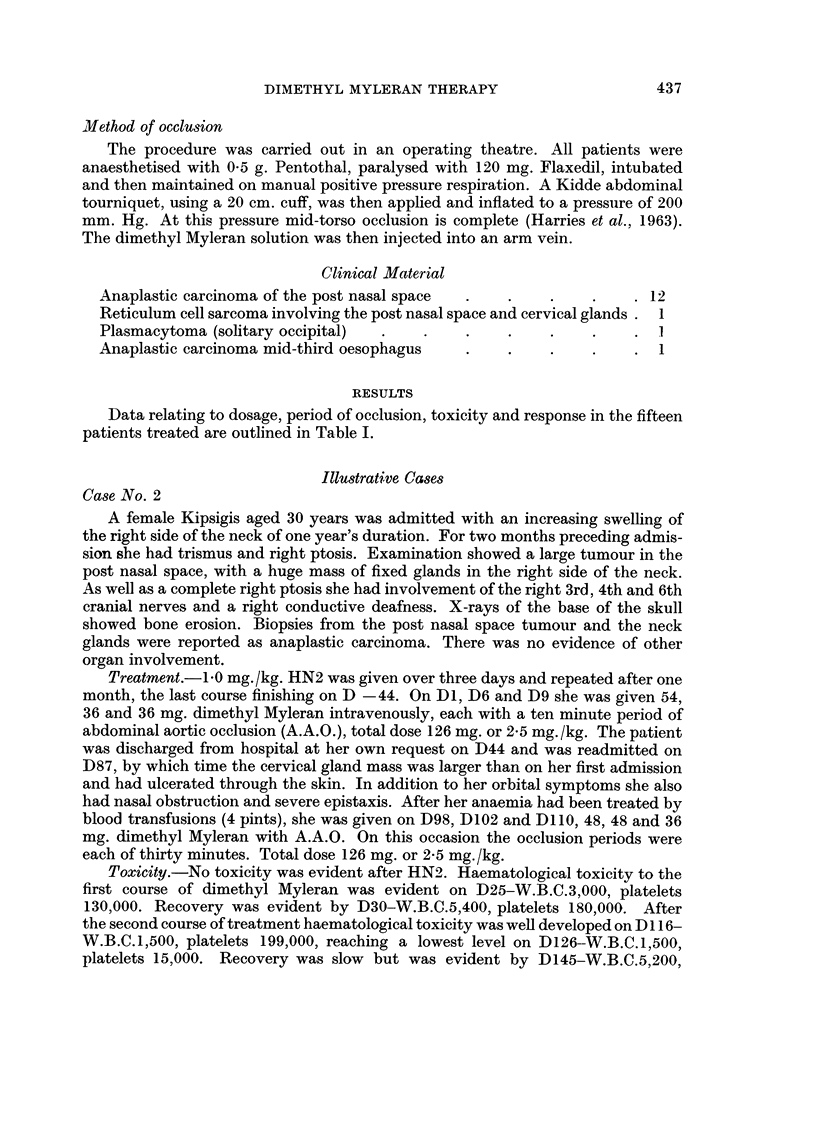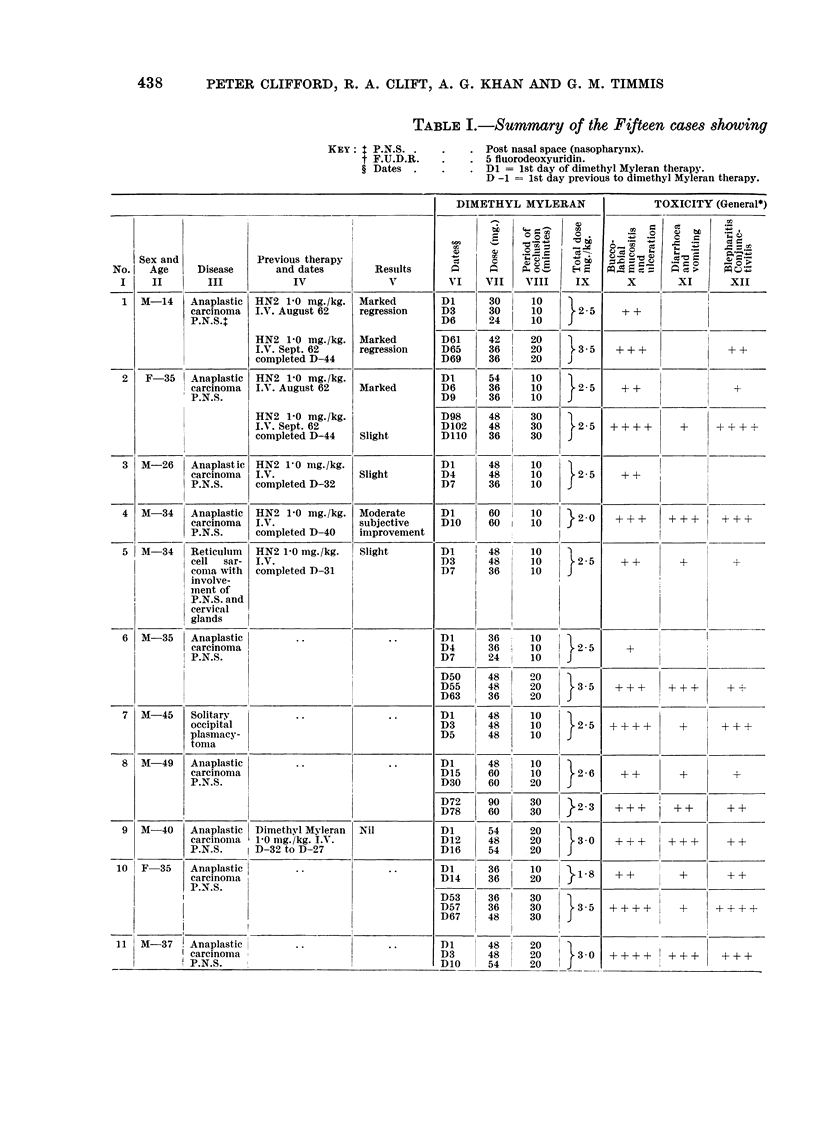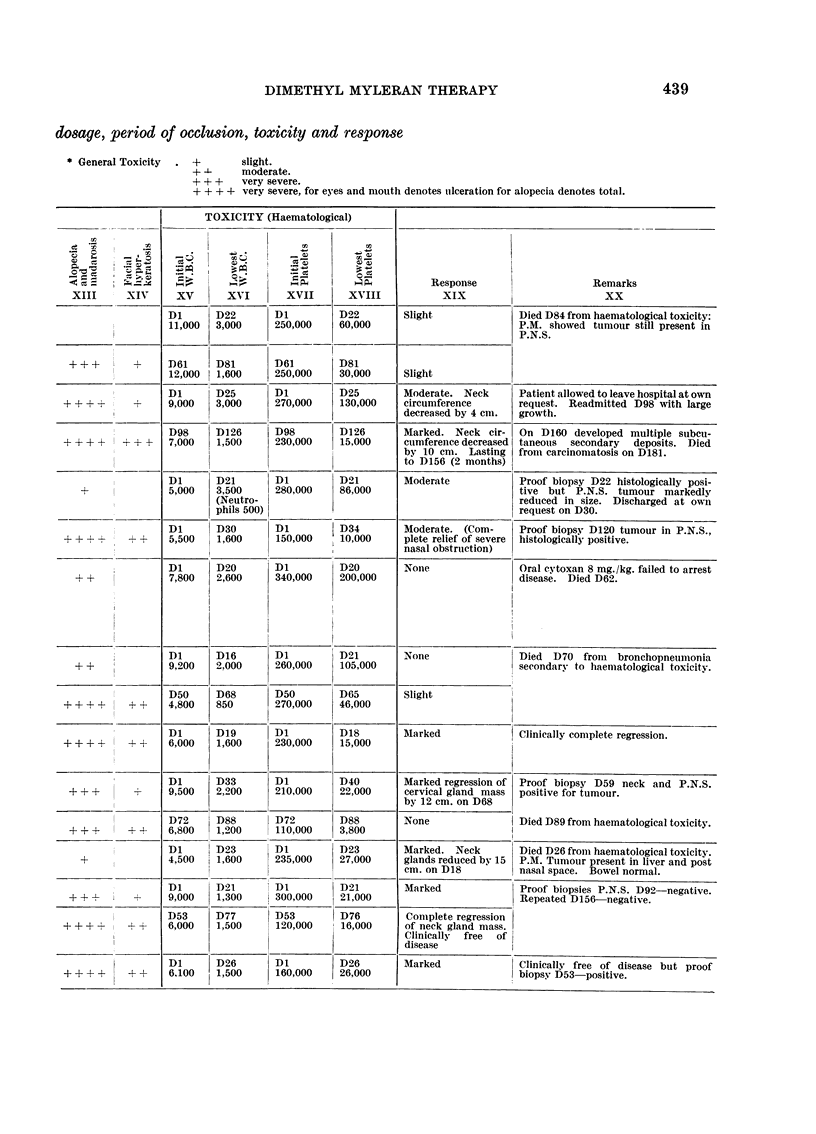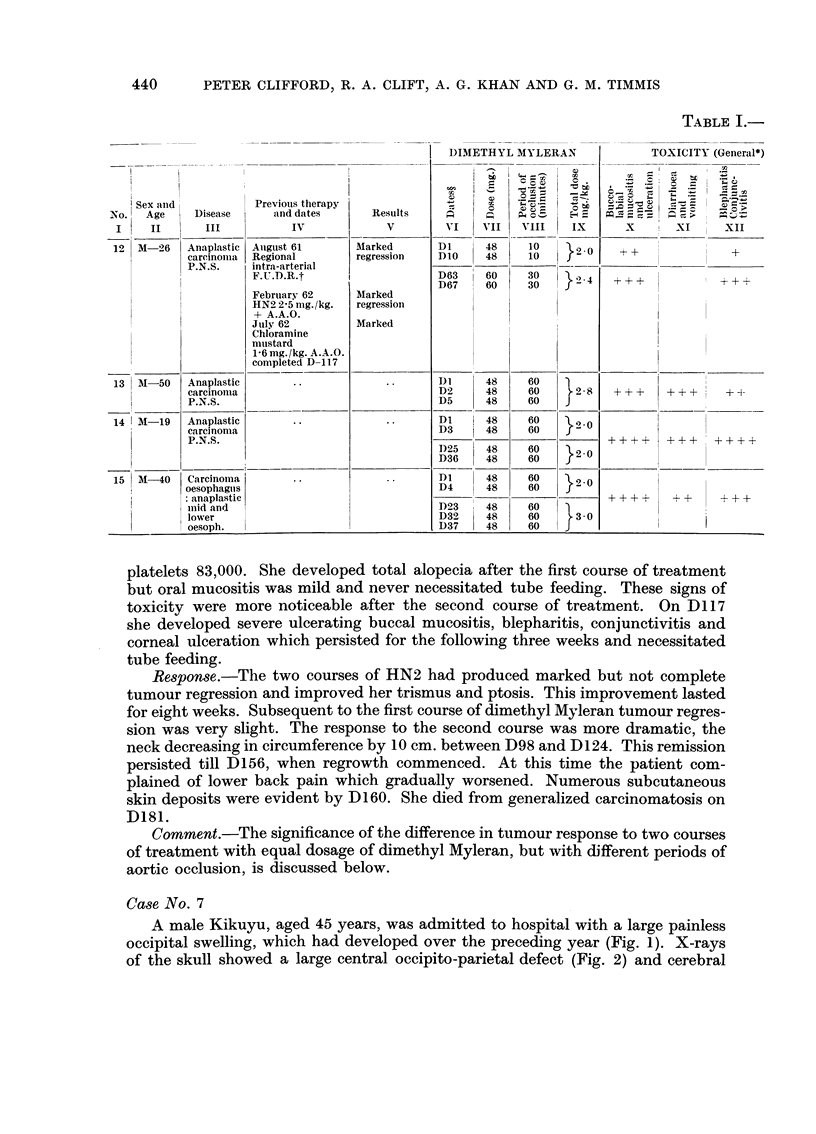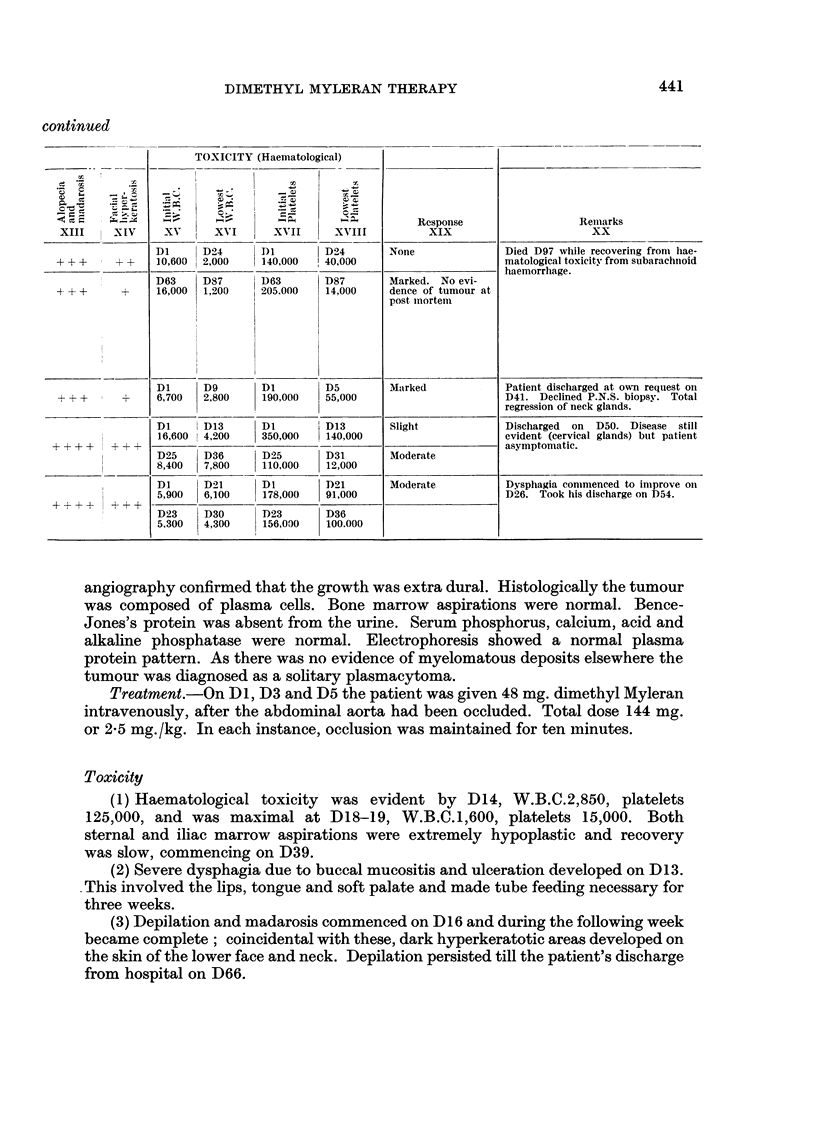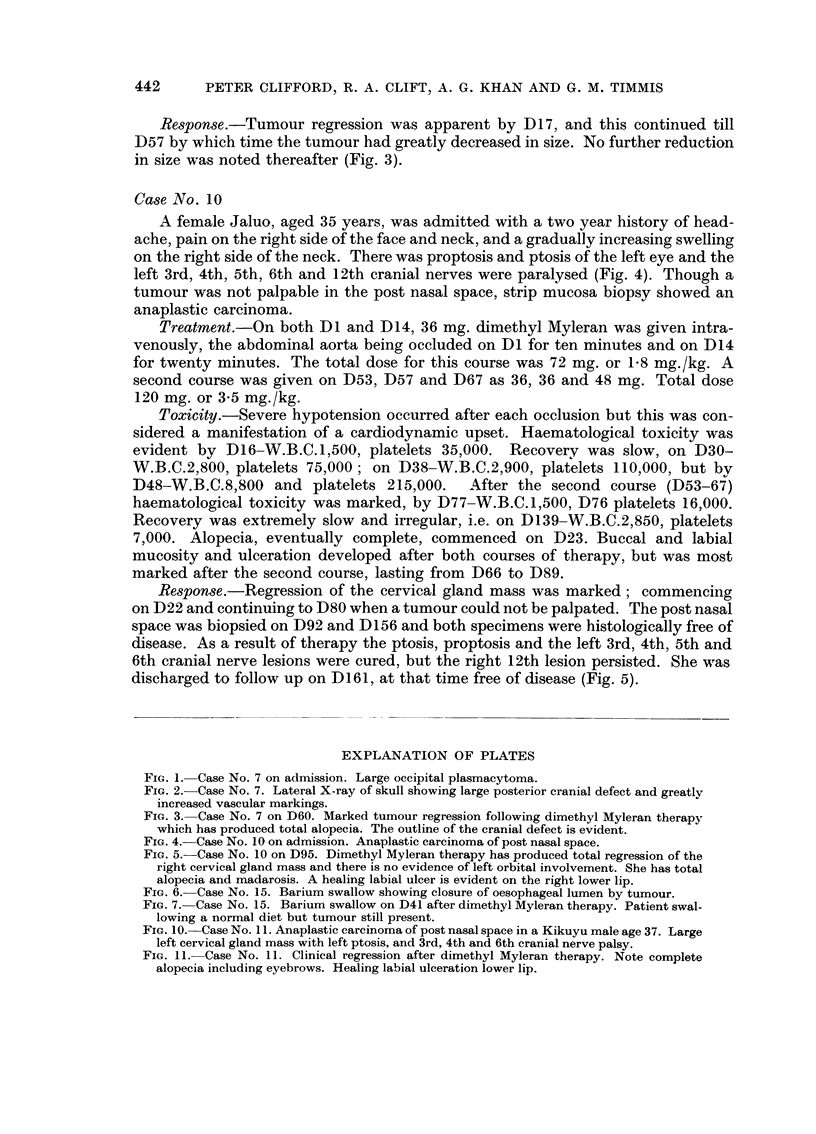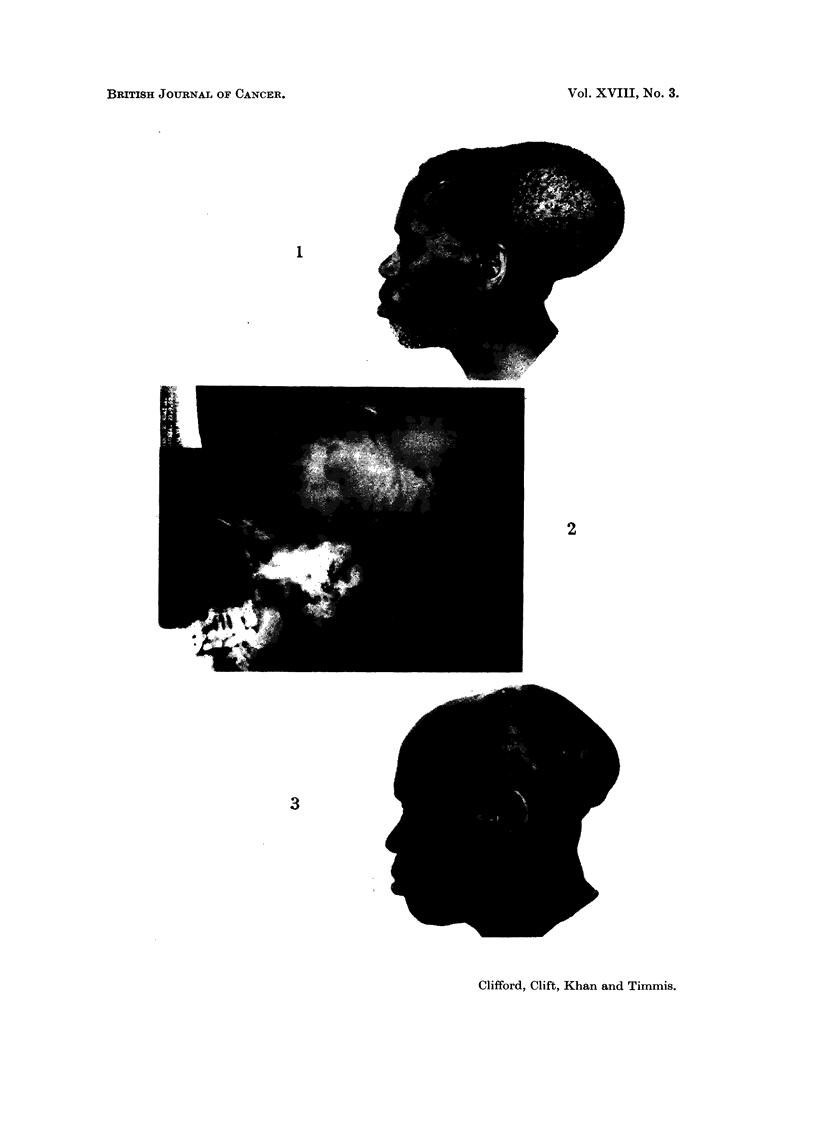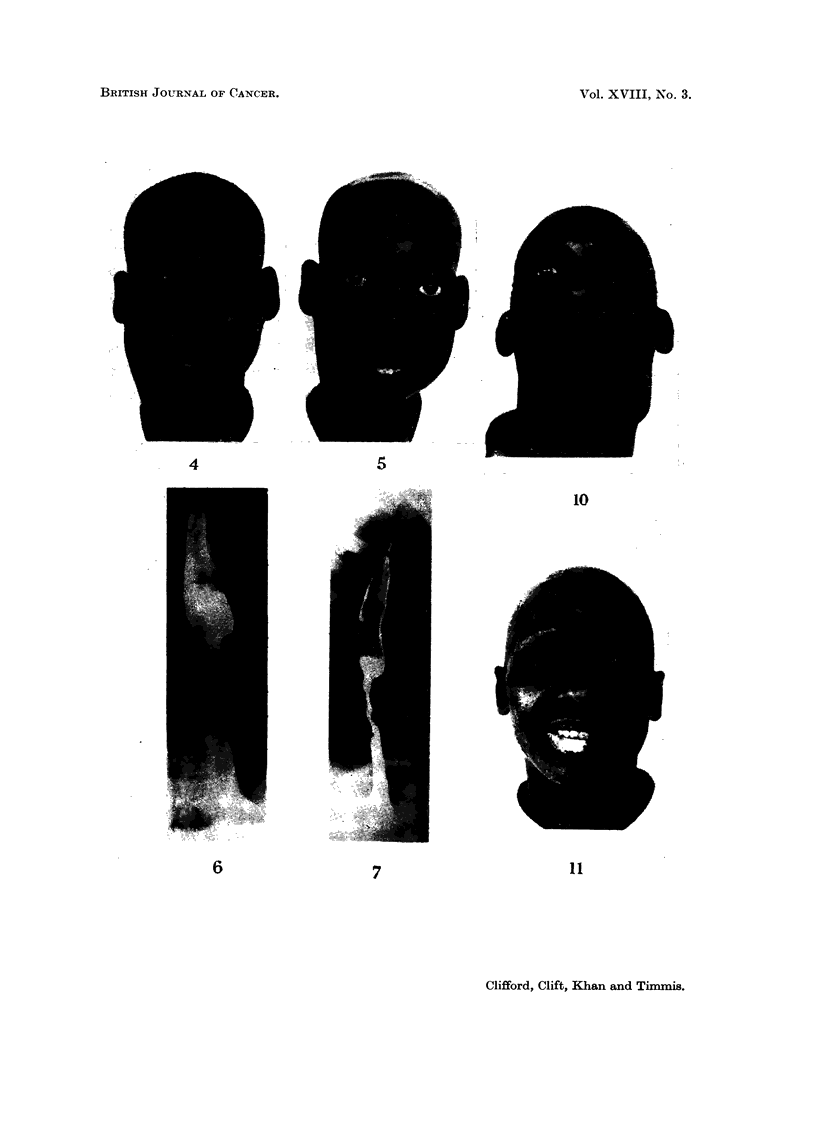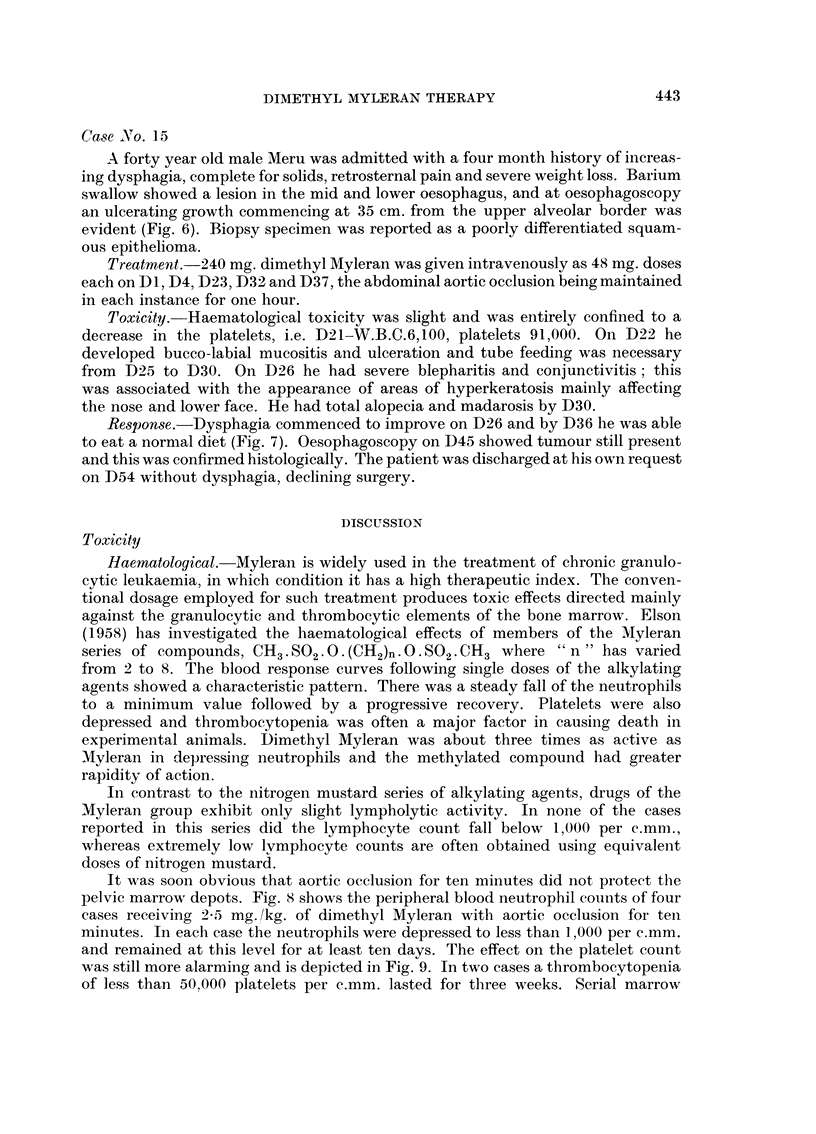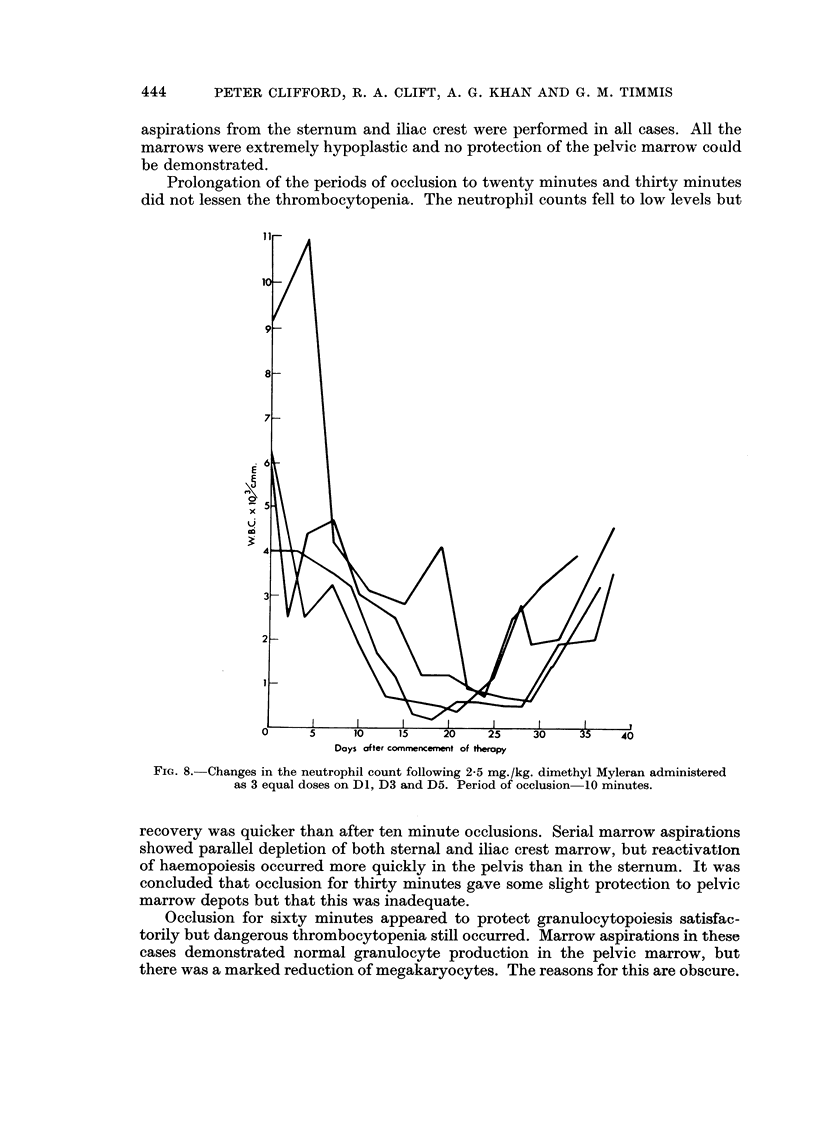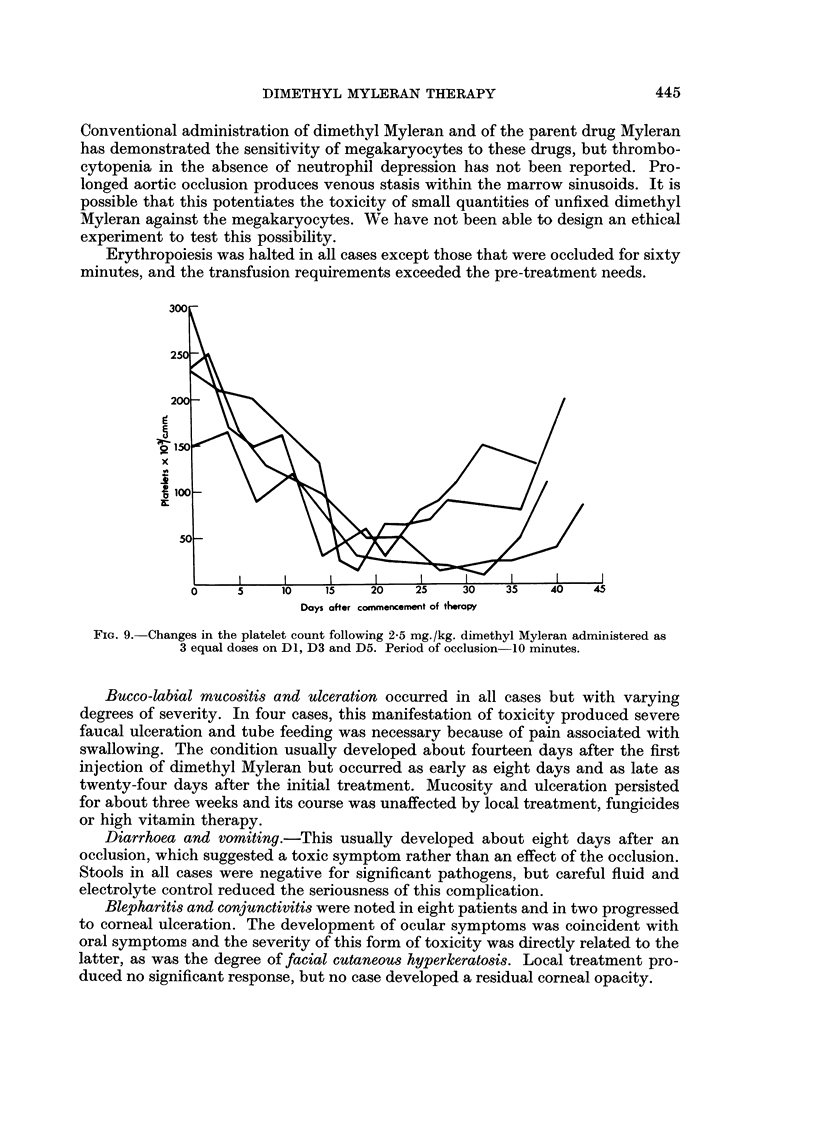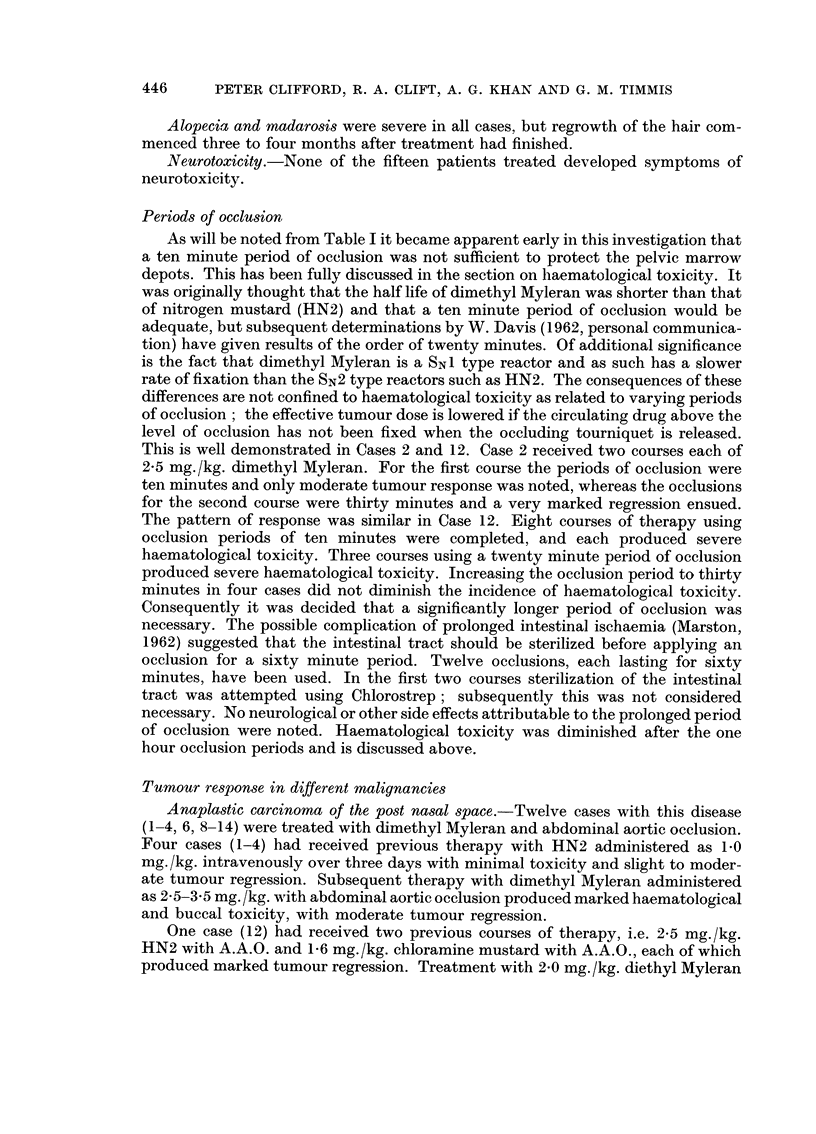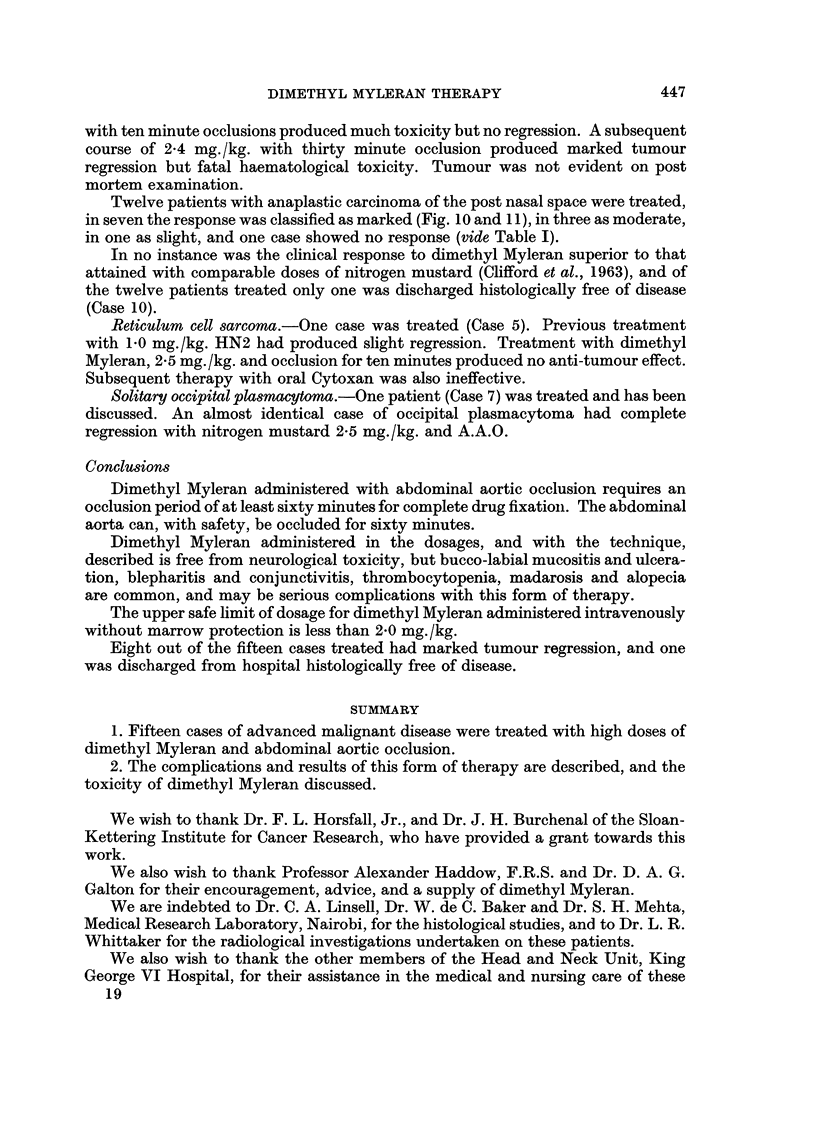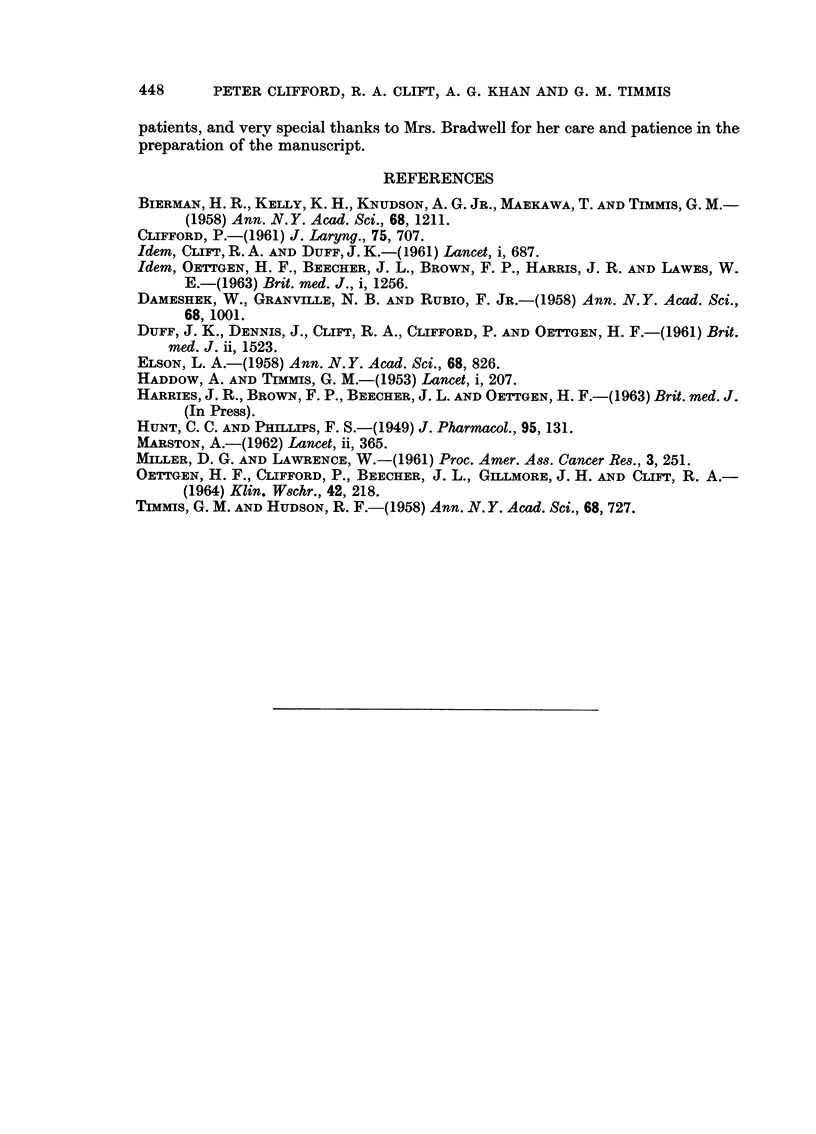# Dimethyl Myleran Therapy Combined with Abdominal Aortic Occlusion

**DOI:** 10.1038/bjc.1964.50

**Published:** 1964-09

**Authors:** Peter Clifford, R. A. Clift, A. G. Khan, G. M. Timmis

## Abstract

**Images:**


### DIMETHYL MYLERAN THERAPY COMBINED WITH ABDOMINAL AORTIC OCCLUSION

PETER CLIFFORD*, R. A. CLIFT†, A. G. KHAN AND G. M. TIMMIS

*From the *Department of Head and Neck Surgery, and †The Medical Research Laboratory, King George VI Hospital, Nairobi, Kenya and the Chester Beatty Research Institute, Institute of Cancer Research : Royal Cancer Hospital, London, S.W.3*

Received for publication July 13, 1964

HADDOW and Timmis (1953) introduced the sulfonoxy butanes as cancer chemotherapeutic agents and one of this group of alkylating drugs, Myleran (1,4-dimethylsulfonoxy butane, $CH_3—SO_2—O—CH_2.CH_2.CH_2.CH_2—O—SO_2—CH_3$) has been found useful in the management of chronic granulocytic leukaemia (Dameshek, Granville and Rubio, 1958). Because of its insolubility Myleran can only be administered orally and its maximal effects on the haemopoietic system require 2–3 weeks to develop.

Timmis and Hudson (1958) produced 1,4-dimethyl sulfonox-1,4-ydimethyl butane (CB. 2348) or dimethyl Myleran :

$$\begin{array}{l} CH_3—SO_2—O—\underset{\displaystyle CH_3}{\underset{|}{CH}}.CH_2.CH_2.\underset{\displaystyle CH_3}{\underset{|}{CH}}.—O—SO_2—CH_3 \end{array}$$

The addition of methyl groups to the terminal carbon atoms in the Myleran molecule changed the mechanism of alkylation from a largely $S_N2$ to an $S_N1$ type and produced a compound of greater solubility with a quicker onset of action. This compound is soluble in warm 95 per cent alcohol, and the solution may subsequently be diluted to a convenient volume with normal saline. Bierman *et al.* (1958) found that this drug in doses of 0·4–0·8 mg./kg. produced remission in chronic granulocytic leukaemia but had no effect in acute myeloid leukaemia or on solid tumours.

*Rationale*

The management of malignant disease of the head and neck area in East Africa presents problems not encountered in more highly developed countries (Clifford, 1961). Radiotherapy is not available, and for patients presenting with growths unsuitable for surgery or for regional arterial infusion therapy, treatment with nitrogen mustard was attempted : initially the recommended pharmacopoeial dose (0·1 mg./kg. body weight, daily for 5 days) was given, but as experience suggested that tumour response was proportional to the dose administered, attention was directed to the development of methods designed to allow larger doses of the drug to be given with safety to the patient. It was found that 2·0 mg./kg. HN2 was tolerated if stored autologous bone marrow was used to compensate for marrow depression (Clifford, Clift and Duff, 1961). Even with this dose effective palliation was limited to the more anaplastic and lymphomatous growths. Higher doses of HN2 caused death due to septicaemia, secondary to gastro-intestinal toxicity before the marrow graft had fully developed. Miller and Lawrence (1961) developed a method of protecting the pelvic bone marrow by temporarily occluding the abdominal aorta, but noted that a dose of 1·2 mg./kg. HN2 produced 8th nerve damage. To avoid this and other cerebral complications, the total dose of HN2 was administered as fractions over a three-week period whilst the pelvic marrow was protected by occluding the abdominal aorta (Duff *et al.*, 1961). By this method 1·5 to 3·0 mg./kg. HN2 was administered without producing fatal bone marrow injury, and it was estimated that the effective tumour dose was slightly less than doubled above the level of occlusion. Subsequent studies (Clifford *et al.*, 1963) showed that 2·5 mg./kg. administered as 0·8, 0·8 and 0·9 mg./kg. over a three-week period produced complete clinical and histological remission of disease in thirteen out of eighteen patients with anaplastic carcinoma of the post nasal space, but only one remained without recurrence for longer than six months. On attempting to increase the dosage to a total of 3·0 mg./kg. over a three-week period it was found that the majority of patients succumbed to cerebral toxicity. Attempts to overcome this barrier were directed along two lines :

(1) Isolating the brain under hypothermia (6 min. at 29·4° C.) by clamping the cranial arteries in the neck. Any HN2 still unfixed before unclamping the cranial arteries could be neutralised by injecting 20 c.c. of a 50 per cent solution of sodium thiosulphate, intravenously. The results and complications of this procedure will be published elsewhere.

(2) By using an alkylating agent which might be less neurotoxic.

(*a*) Chloramine mustard (methyl-2-chloroethyl-ethylenimonium) a metabolite of nitrogen mustard was prepared according to the method of Hunt and Philips (1949). This compound was found to be quite as neurotoxic, and clinically less effective, in comparable doses to HN2. Of twenty patients with anaplastic carcinoma of the post nasal space treated with 2·4–3·0 mg./kg. chloramine mustard and abdominal aortic occlusion, ten patients developed a life-threatening degree of toxicity, and six of these succumbed to neurotoxicity. Of the fourteen patients who survived, only three were free of recurrence for longer than one month (Oettgen *et al.*, 1964).

(*b*) Dimethyl Myleran probably has a different mechanism of action from the nitrogen mustards since it does not alkylate nucleophilic centres in aqueous media at physiological pH because it reacts with water far more readily. The most likely mechanism therefore is that it acts in the lipid phase, where the chances of reacting with an important nucleophilic centre such as phosphate in preference to water, would be much greater. A possible site of action could well be in the lipid protein interphases within the cytoplasm which are thought to play an important part in protein synthesis. In addition, Myleran has shown no neurotoxicity in clinical use and since dimethyl Myleran has behaved similarly in a small number of clinical experiments we concluded that this drug was now worth trying. This paper describes the complications and results in treating fifteen patients with advanced malignant disease of the head and neck with dimethyl Myleran and abdominal aortic occlusion.

### *Preparation of dimethyl Myleran solution*

In all cases a freshly prepared solution was used. 60 mg. of dimethyl Myleran were dissolved in 5 ml. of ethyl alcohol, warmed to 50° C., and an aliquot of this solution was diluted 1 : 5 with sterile isotonic saline.

*Method of occlusion*

The procedure was carried out in an operating theatre. All patients were anaesthetised with 0·5 g. Pentothal, paralysed with 120 mg. Flaxedil, intubated and then maintained on manual positive pressure respiration. A Kidde abdominal tourniquet, using a 20 cm. cuff, was then applied and inflated to a pressure of 200 mm. Hg. At this pressure mid-torso occlusion is complete (Harries *et al.*, 1963). The dimethyl Myleran solution was then injected into an arm vein.

### *Clinical Material*

| | |
|---|---|
| Anaplastic carcinoma of the post nasal space . . . . . . | 12 |
| Reticulum cell sarcoma involving the post nasal space and cervical glands . | 1 |
| Plasmacytoma (solitary occipital) . . . . . . . . . | 1 |
| Anaplastic carcinoma mid-third oesophagus . . . . . . | 1 |

### RESULTS

Data relating to dosage, period of occlusion, toxicity and response in the fifteen patients treated are outlined in Table I.

### *Illustrative Cases*

*Case No.* 2

A female Kipsigis aged 30 years was admitted with an increasing swelling of the right side of the neck of one year's duration. For two months preceding admission she had trismus and right ptosis. Examination showed a large tumour in the post nasal space, with a huge mass of fixed glands in the right side of the neck. As well as a complete right ptosis she had involvement of the right 3rd, 4th and 6th cranial nerves and a right conductive deafness. X-rays of the base of the skull showed bone erosion. Biopsies from the post nasal space tumour and the neck glands were reported as anaplastic carcinoma. There was no evidence of other organ involvement.

*Treatment.*—1·0 mg./kg. HN2 was given over three days and repeated after one month, the last course finishing on D −44. On D1, D6 and D9 she was given 54, 36 and 36 mg. dimethyl Myleran intravenously, each with a ten minute period of abdominal aortic occlusion (A.A.O.), total dose 126 mg. or 2·5 mg./kg. The patient was discharged from hospital at her own request on D44 and was readmitted on D87, by which time the cervical gland mass was larger than on her first admission and had ulcerated through the skin. In addition to her orbital symptoms she also had nasal obstruction and severe epistaxis. After her anaemia had been treated by blood transfusions (4 pints), she was given on D98, D102 and D110, 48, 48 and 36 mg. dimethyl Myleran with A.A.O. On this occasion the occlusion periods were each of thirty minutes. Total dose 126 mg. or 2·5 mg./kg.

*Toxicity.*—No toxicity was evident after HN2. Haematological toxicity to the first course of dimethyl Myleran was evident on D25–W.B.C.3,000, platelets 130,000. Recovery was evident by D30–W.B.C.5,400, platelets 180,000. After the second course of treatment haematological toxicity was well developed on D116–W.B.C.1,500, platelets 199,000, reaching a lowest level on D126–W.B.C.1,500, platelets 15,000. Recovery was slow but was evident by D145–W.B.C.5,200,

TABLE I.—*Summary of the Fifteen cases showing*

KEY : ‡ P.N.S. . . . Post nasal space (nasopharynx).

† F.U.D.R. . . . 5 fluorodeoxyuridin.

§ Dates . . . D1 = 1st day of dimethyl Myleran therapy.

D -1 = 1st day previous to dimethyl Myleran therapy.

| | | | | | DIMETHYL MYLERAN | | | | TOXICITY (General*) | | |
|---|---|---|---|---|---|---|---|---|---|---|---|
| No.<br>I | Sex and Age<br>II | Disease<br>III | Previous therapy and dates<br>IV | Results<br>V | Dates§<br>VI | Dose (mg.)<br>VII | Period of occlusion (minutes)<br>VIII | Total dose mg./kg.<br>IX | Bucco-labial mucositis and ulceration<br>X | Diarrhoea and vomiting<br>XI | Blepharitis Conjunctivitis<br>XII |
| 1 | M—14 | Anaplastic carcinoma P.N.S.‡ | HN2 1·0 mg./kg. I.V. August 62 | Marked regression | D1<br>D3<br>D6 | 30<br>30<br>24 | 10<br>10<br>10 | 2·5 | + + | | |
| | | | HN2 1·0 mg./kg. I.V. Sept. 62 completed D–44 | Marked regression | D61<br>D65<br>D69 | 42<br>36<br>36 | 20<br>20<br>20 | 3·5 | + + + | | + + |
| 2 | F—35 | Anaplastic carcinoma P.N.S. | HN2 1·0 mg./kg. I.V. August 62 | Marked | D1<br>D6<br>D9 | 54<br>36<br>36 | 10<br>10<br>10 | 2·5 | + + | | + |
| | | | HN2 1·0 mg./kg. I.V. Sept. 62 completed D–44 | Slight | D98<br>D102<br>D110 | 48<br>48<br>36 | 30<br>30<br>30 | 2·5 | + + + + | + | + + + + |
| 3 | M—26 | Anaplastic carcinoma P.N.S. | HN2 1·0 mg./kg. I.V. completed D–32 | Slight | D1<br>D4<br>D7 | 48<br>48<br>36 | 10<br>10<br>10 | 2·5 | + + | | |
| 4 | M—34 | Anaplastic carcinoma P.N.S. | HN2 1·0 mg./kg. I.V. completed D–40 | Moderate subjective improvement | D1<br>D10 | 60<br>60 | 10<br>10 | 2·0 | + + + | + + + | + + + |
| 5 | M—34 | Reticulum cell sarcoma with involvement of P.N.S. and cervical glands | HN2 1·0 mg./kg. I.V. completed D–31 | Slight | D1<br>D3<br>D7 | 48<br>48<br>36 | 10<br>10<br>10 | 2·5 | + + | + | + |
| 6 | M—35 | Anaplastic carcinoma P.N.S. | . . | . . | D1<br>D4<br>D7 | 36<br>36<br>24 | 10<br>10<br>10 | 2·5 | + | | |
| | | | | | D50<br>D55<br>D63 | 48<br>48<br>36 | 20<br>20<br>20 | 3·5 | + + + | + + + | + + |
| 7 | M—45 | Solitary occipital plasmacytoma | . . | . . | D1<br>D3<br>D5 | 48<br>48<br>48 | 10<br>10<br>10 | 2·5 | + + + + | + | + + + |
| 8 | M—49 | Anaplastic carcinoma P.N.S. | . . | . . | D1<br>D15<br>D30 | 48<br>60<br>60 | 10<br>10<br>20 | 2·6 | + + | + | + |
| | | | | | D72<br>D78 | 90<br>60 | 30<br>30 | 2·3 | + + + | + + | + + |
| 9 | M—40 | Anaplastic carcinoma P.N.S. | Dimethyl Myleran 1·0 mg./kg. I.V. D–32 to D–27 | Nil | D1<br>D12<br>D16 | 54<br>48<br>54 | 20<br>20<br>20 | 3·0 | + + + | + + + | + + |
| 10 | F—35 | Anaplastic carcinoma P.N.S. | . . | . . | D1<br>D14 | 36<br>36 | 10<br>20 | 1·8 | + + | + | + + |
| | | | | | D53<br>D57<br>D67 | 36<br>36<br>48 | 30<br>30<br>30 | 3·5 | + + + + | + | + + + + |
| 11 | M—37 | Anaplastic carcinoma P.N.S. | . . | . . | D1<br>D3<br>D10 | 48<br>48<br>54 | 20<br>20<br>20 | 3·0 | + + + + | + + + | + + + |

*dosage, period of occlusion, toxicity and response*

* General Toxicity . + slight.
++ moderate.
+++ very severe.
++++ very severe, for eyes and mouth denotes ulceration for alopecia denotes total.

| | | TOXICITY (Haematological) | | | | | |
|---|---|---|---|---|---|---|---|
| Alopecia and madarosis XIII | Facial hyper-keratosis XIV | Initial W.B.C. XV | Lowest W.B.C. XVI | Initial Platelets XVII | Lowest Platelets XVIII | Response XIX | Remarks XX |
| | | D1 11,000 | D22 3,000 | D1 250,000 | D22 60,000 | Slight | Died D84 from haematological toxicity: P.M. showed tumour still present in P.N.S. |
| +++ | + | D61 12,000 | D81 1,600 | D61 250,000 | D81 30,000 | Slight | |
| ++++ | + | D1 9,000 | D25 3,000 | D1 270,000 | D25 130,000 | Moderate. Neck circumference decreased by 4 cm. | Patient allowed to leave hospital at own request. Readmitted D98 with large growth. |
| ++++ | +++ | D98 7,000 | D126 1,500 | D98 230,000 | D126 15,000 | Marked. Neck circumference decreased by 10 cm. Lasting to D156 (2 months) | On D160 developed multiple subcutaneous secondary deposits. Died from carcinomatosis on D181. |
| + | | D1 5,000 | D21 3,500 (Neutrophils 500) | D1 280,000 | D21 86,000 | Moderate | Proof biopsy D22 histologically positive but P.N.S. tumour markedly reduced in size. Discharged at own request on D30. |
| ++++ | ++ | D1 5,500 | D30 1,600 | D1 150,000 | D34 10,000 | Moderate. (Complete relief of severe nasal obstruction) | Proof biopsy D120 tumour in P.N.S., histologically positive. |
| ++ | | D1 7,800 | D20 2,600 | D1 340,000 | D20 200,000 | None | Oral cytoxan 8 mg./kg. failed to arrest disease. Died D62. |
| ++ | | D1 9,200 | D16 2,000 | D1 260,000 | D21 105,000 | None | Died D70 from bronchopneumonia secondary to haematological toxicity. |
| ++++ | ++ | D50 4,800 | D68 850 | D50 270,000 | D65 46,000 | Slight | |
| ++++ | ++ | D1 6,000 | D19 1,600 | D1 230,000 | D18 15,000 | Marked | Clinically complete regression. |
| +++ | + | D1 9,500 | D33 2,200 | D1 210,000 | D40 22,000 | Marked regression of cervical gland mass by 12 cm. on D68 | Proof biopsy D59 neck and P.N.S. positive for tumour. |
| +++ | ++ | D72 6,800 | D88 1,200 | D72 110,000 | D88 3,800 | None | Died D89 from haematological toxicity. |
| + | | D1 4,500 | D23 1,600 | D1 235,000 | D23 27,000 | Marked. Neck glands reduced by 15 cm. on D18 | Died D26 from haematological toxicity. P.M. Tumour present in liver and post nasal space. Bowel normal. |
| +++ | + | D1 9,000 | D21 1,300 | D1 300,000 | D21 21,000 | Marked | Proof biopsies P.N.S. D92—negative. Repeated D156—negative. |
| ++++ | ++ | D53 6,000 | D77 1,500 | D53 120,000 | D76 16,000 | Complete regression of neck gland mass. Clinically free of disease | |
| ++++ | ++ | D1 6,100 | D26 1,500 | D1 160,000 | D26 26,000 | Marked | Clinically free of disease but proof biopsy D53—positive. |

TABLE I.—

| | | | | | DIMETHYL MYLERAN | | | | TOXICITY (General*) | | |
|---|---|---|---|---|---|---|---|---|---|---|---|
| No.<br>I | Sex and Age<br>II | Disease<br>III | Previous therapy and dates<br>IV | Results<br>V | Dates§<br>VI | Dose (mg.)<br>VII | Period of occlusion (minutes)<br>VIII | Total dose mg./kg.<br>IX | Bucco-labial mucositis and ulceration<br>X | Diarrhoea and vomiting<br>XI | Blepharitis Conjunc-tivitis<br>XII |
| 12 | M—26 | Anaplastic carcinoma P.N.S. | August 61 Regional intra-arterial F.U.D.R.† | Marked regression | D1<br>D10 | 48<br>48 | 10<br>10 | 2·0 | ++ | | + |
| | | | February 62 HN2 2·5 mg./kg. + A.A.O.<br>July 62 Chloramine mustard 1·6 mg./kg. A.A.O. completed D-117 | Marked regression<br>Marked | D63<br>D67 | 60<br>60 | 30<br>30 | 2·4 | +++ | | +++ |
| 13 | M—50 | Anaplastic carcinoma P.N.S. | .. | .. | D1<br>D2<br>D5 | 48<br>48<br>48 | 60<br>60<br>60 | 2·8 | +++ | +++ | ++ |
| 14 | M—19 | Anaplastic carcinoma P.N.S. | .. | .. | D1<br>D3 | 48<br>48 | 60<br>60 | 2·0 | ++++ | +++ | ++++ |
| | | | | | D25<br>D36 | 48<br>48 | 60<br>60 | 2·0 | | | |
| 15 | M—40 | Carcinoma oesophagus : anaplastic mid and lower oesoph. | .. | .. | D1<br>D4 | 48<br>48 | 60<br>60 | 2·0 | ++++ | ++ | +++ |
| | | | | | D23<br>D32<br>D37 | 48<br>48<br>48 | 60<br>60<br>60 | 3·0 | | | |

platelets 83,000. She developed total alopecia after the first course of treatment but oral mucositis was mild and never necessitated tube feeding. These signs of toxicity were more noticeable after the second course of treatment. On D117 she developed severe ulcerating buccal mucositis, blepharitis, conjunctivitis and corneal ulceration which persisted for the following three weeks and necessitated tube feeding.

*Response.*—The two courses of HN2 had produced marked but not complete tumour regression and improved her trismus and ptosis. This improvement lasted for eight weeks. Subsequent to the first course of dimethyl Myleran tumour regression was very slight. The response to the second course was more dramatic, the neck decreasing in circumference by 10 cm. between D98 and D124. This remission persisted till D156, when regrowth commenced. At this time the patient complained of lower back pain which gradually worsened. Numerous subcutaneous skin deposits were evident by D160. She died from generalized carcinomatosis on D181.

*Comment.*—The significance of the difference in tumour response to two courses of treatment with equal dosage of dimethyl Myleran, but with different periods of aortic occlusion, is discussed below.

*Case No.* 7

A male Kikuyu, aged 45 years, was admitted to hospital with a large painless occipital swelling, which had developed over the preceding year (Fig. 1). X-rays of the skull showed a large central occipito-parietal defect (Fig. 2) and cerebral

*continued*

| | | TOXICITY (Haematological) | | | | | |
|---|---|---|---|---|---|---|---|
| Alopecia and madarosis XIII | Facial hyper-keratosis XIV | Initial W.B.C. XV | Lowest W.B.C. XVI | Initial Platelets XVII | Lowest Platelets XVIII | Response XIX | Remarks XX |
| + + + | + + | D1 10,600 | D24 2,000 | D1 140,000 | D24 40,000 | None | Died D97 while recovering from haematological toxicity from subarachnoid haemorrhage. |
| + + + | + | D63 16,000 | D87 1,200 | D63 205,000 | D87 14,000 | Marked. No evidence of tumour at post mortem | |
| + + + | + | D1 6,700 | D9 2,800 | D1 190,000 | D5 55,000 | Marked | Patient discharged at own request on D41. Declined P.N.S. biopsy. Total regression of neck glands. |
| + + + + | + + + | D1 16,600 | D13 4,200 | D1 350,000 | D13 140,000 | Slight | Discharged on D50. Disease still evident (cervical glands) but patient asymptomatic. |
| | | D25 8,400 | D36 7,800 | D25 110,000 | D31 12,000 | Moderate | |
| + + + + | + + + | D1 5,900 | D21 6,100 | D1 178,000 | D21 91,000 | Moderate | Dysphagia commenced to improve on D26. Took his discharge on D54. |
| | | D23 5,300 | D30 4,300 | D23 156,000 | D36 100,000 | | |

angiography confirmed that the growth was extra dural. Histologically the tumour was composed of plasma cells. Bone marrow aspirations were normal. Bence-Jones's protein was absent from the urine. Serum phosphorus, calcium, acid and alkaline phosphatase were normal. Electrophoresis showed a normal plasma protein pattern. As there was no evidence of myelomatous deposits elsewhere the tumour was diagnosed as a solitary plasmacytoma.

*Treatment*.—On D1, D3 and D5 the patient was given 48 mg. dimethyl Myleran intravenously, after the abdominal aorta had been occluded. Total dose 144 mg. or 2·5 mg./kg. In each instance, occlusion was maintained for ten minutes.

*Toxicity*

(1) Haematological toxicity was evident by D14, W.B.C.2,850, platelets 125,000, and was maximal at D18–19, W.B.C.1,600, platelets 15,000. Both sternal and iliac marrow aspirations were extremely hypoplastic and recovery was slow, commencing on D39.

(2) Severe dysphagia due to buccal mucositis and ulceration developed on D13. This involved the lips, tongue and soft palate and made tube feeding necessary for three weeks.

(3) Depilation and madarosis commenced on D16 and during the following week became complete ; coincidental with these, dark hyperkeratotic areas developed on the skin of the lower face and neck. Depilation persisted till the patient's discharge from hospital on D66.

*Response*.—Tumour regression was apparent by D17, and this continued till D57 by which time the tumour had greatly decreased in size. No further reduction in size was noted thereafter (Fig. 3).

*Case No*. 10

A female Jaluo, aged 35 years, was admitted with a two year history of headache, pain on the right side of the face and neck, and a gradually increasing swelling on the right side of the neck. There was proptosis and ptosis of the left eye and the left 3rd, 4th, 5th, 6th and 12th cranial nerves were paralysed (Fig. 4). Though a tumour was not palpable in the post nasal space, strip mucosa biopsy showed an anaplastic carcinoma.

*Treatment*.—On both D1 and D14, 36 mg. dimethyl Myleran was given intravenously, the abdominal aorta being occluded on D1 for ten minutes and on D14 for twenty minutes. The total dose for this course was 72 mg. or 1·8 mg./kg. A second course was given on D53, D57 and D67 as 36, 36 and 48 mg. Total dose 120 mg. or 3·5 mg./kg.

*Toxicity*.—Severe hypotension occurred after each occlusion but this was considered a manifestation of a cardiodynamic upset. Haematological toxicity was evident by D16–W.B.C.1,500, platelets 35,000. Recovery was slow, on D30–W.B.C.2,800, platelets 75,000 ; on D38–W.B.C.2,900, platelets 110,000, but by D48–W.B.C.8,800 and platelets 215,000. After the second course (D53–67) haematological toxicity was marked, by D77–W.B.C.1,500, D76 platelets 16,000. Recovery was extremely slow and irregular, i.e. on D139–W.B.C.2,850, platelets 7,000. Alopecia, eventually complete, commenced on D23. Buccal and labial mucosity and ulceration developed after both courses of therapy, but was most marked after the second course, lasting from D66 to D89.

*Response*.—Regression of the cervical gland mass was marked ; commencing on D22 and continuing to D80 when a tumour could not be palpated. The post nasal space was biopsied on D92 and D156 and both specimens were histologically free of disease. As a result of therapy the ptosis, proptosis and the left 3rd, 4th, 5th and 6th cranial nerve lesions were cured, but the right 12th lesion persisted. She was discharged to follow up on D161, at that time free of disease (Fig. 5).

---

EXPLANATION OF PLATES

FIG. 1.—Case No. 7 on admission. Large occipital plasmacytoma.

FIG. 2.—Case No. 7. Lateral X-ray of skull showing large posterior cranial defect and greatly increased vascular markings.

FIG. 3.—Case No. 7 on D60. Marked tumour regression following dimethyl Myleran therapy which has produced total alopecia. The outline of the cranial defect is evident.

FIG. 4.—Case No. 10 on admission. Anaplastic carcinoma of post nasal space.

FIG. 5.—Case No. 10 on D95. Dimethyl Myleran therapy has produced total regression of the right cervical gland mass and there is no evidence of left orbital involvement. She has total alopecia and madarosis. A healing labial ulcer is evident on the right lower lip.

FIG. 6.—Case No. 15. Barium swallow showing closure of oesophageal lumen by tumour.

FIG. 7.—Case No. 15. Barium swallow on D41 after dimethyl Myleran therapy. Patient swallowing a normal diet but tumour still present.

FIG. 10.—Case No. 11. Anaplastic carcinoma of post nasal space in a Kikuyu male age 37. Large left cervical gland mass with left ptosis, and 3rd, 4th and 6th cranial nerve palsy.

FIG. 11.—Case No. 11. Clinical regression after dimethyl Myleran therapy. Note complete alopecia including eyebrows. Healing labial ulceration lower lip.

1

2

3

4

5

10

6

7

11

*Case No.* 15

A forty year old male Meru was admitted with a four month history of increasing dysphagia, complete for solids, retrosternal pain and severe weight loss. Barium swallow showed a lesion in the mid and lower oesophagus, and at oesophagoscopy an ulcerating growth commencing at 35 cm. from the upper alveolar border was evident (Fig. 6). Biopsy specimen was reported as a poorly differentiated squamous epithelioma.

*Treatment.*—240 mg. dimethyl Myleran was given intravenously as 48 mg. doses each on D1, D4, D23, D32 and D37, the abdominal aortic occlusion being maintained in each instance for one hour.

*Toxicity.*—Haematological toxicity was slight and was entirely confined to a decrease in the platelets, i.e. D21–W.B.C.6,100, platelets 91,000. On D22 he developed bucco-labial mucositis and ulceration and tube feeding was necessary from D25 to D30. On D26 he had severe blepharitis and conjunctivitis; this was associated with the appearance of areas of hyperkeratosis mainly affecting the nose and lower face. He had total alopecia and madarosis by D30.

*Response.*—Dysphagia commenced to improve on D26 and by D36 he was able to eat a normal diet (Fig. 7). Oesophagoscopy on D45 showed tumour still present and this was confirmed histologically. The patient was discharged at his own request on D54 without dysphagia, declining surgery.

### DISCUSSION

### *Toxicity*

*Haematological.*—Myleran is widely used in the treatment of chronic granulocytic leukaemia, in which condition it has a high therapeutic index. The conventional dosage employed for such treatment produces toxic effects directed mainly against the granulocytic and thrombocytic elements of the bone marrow. Elson (1958) has investigated the haematological effects of members of the Myleran series of compounds, $CH_3.SO_2.O.(CH_2)_n.O.SO_2.CH_3$ where "n" has varied from 2 to 8. The blood response curves following single doses of the alkylating agents showed a characteristic pattern. There was a steady fall of the neutrophils to a minimum value followed by a progressive recovery. Platelets were also depressed and thrombocytopenia was often a major factor in causing death in experimental animals. Dimethyl Myleran was about three times as active as Myleran in depressing neutrophils and the methylated compound had greater rapidity of action.

In contrast to the nitrogen mustard series of alkylating agents, drugs of the Myleran group exhibit only slight lympholytic activity. In none of the cases reported in this series did the lymphocyte count fall below 1,000 per c.mm., whereas extremely low lymphocyte counts are often obtained using equivalent doses of nitrogen mustard.

It was soon obvious that aortic occlusion for ten minutes did not protect the pelvic marrow depots. Fig. 8 shows the peripheral blood neutrophil counts of four cases receiving 2·5 mg./kg. of dimethyl Myleran with aortic occlusion for ten minutes. In each case the neutrophils were depressed to less than 1,000 per c.mm. and remained at this level for at least ten days. The effect on the platelet count was still more alarming and is depicted in Fig. 9. In two cases a thrombocytopenia of less than 50,000 platelets per c.mm. lasted for three weeks. Serial marrow aspirations from the sternum and iliac crest were performed in all cases. All the marrows were extremely hypoplastic and no protection of the pelvic marrow could be demonstrated.

Prolongation of the periods of occlusion to twenty minutes and thirty minutes did not lessen the thrombocytopenia. The neutrophil counts fell to low levels but

FIG. 8.—Changes in the neutrophil count following 2·5 mg./kg. dimethyl Myleran administered as 3 equal doses on D1, D3 and D5. Period of occlusion—10 minutes.

recovery was quicker than after ten minute occlusions. Serial marrow aspirations showed parallel depletion of both sternal and iliac crest marrow, but reactivation of haemopoiesis occurred more quickly in the pelvis than in the sternum. It was concluded that occlusion for thirty minutes gave some slight protection to pelvic marrow depots but that this was inadequate.

Occlusion for sixty minutes appeared to protect granulocytopoiesis satisfactorily but dangerous thrombocytopenia still occurred. Marrow aspirations in these cases demonstrated normal granulocyte production in the pelvic marrow, but there was a marked reduction of megakaryocytes. The reasons for this are obscure.

Conventional administration of dimethyl Myleran and of the parent drug Myleran has demonstrated the sensitivity of megakaryocytes to these drugs, but thrombocytopenia in the absence of neutrophil depression has not been reported. Prolonged aortic occlusion produces venous stasis within the marrow sinusoids. It is possible that this potentiates the toxicity of small quantities of unfixed dimethyl Myleran against the megakaryocytes. We have not been able to design an ethical experiment to test this possibility.

Erythropoiesis was halted in all cases except those that were occluded for sixty minutes, and the transfusion requirements exceeded the pre-treatment needs.

FIG. 9.—Changes in the platelet count following 2·5 mg./kg. dimethyl Myleran administered as 3 equal doses on D1, D3 and D5. Period of occlusion—10 minutes.

*Bucco-labial mucositis and ulceration* occurred in all cases but with varying degrees of severity. In four cases, this manifestation of toxicity produced severe faucal ulceration and tube feeding was necessary because of pain associated with swallowing. The condition usually developed about fourteen days after the first injection of dimethyl Myleran but occurred as early as eight days and as late as twenty-four days after the initial treatment. Mucosity and ulceration persisted for about three weeks and its course was unaffected by local treatment, fungicides or high vitamin therapy.

*Diarrhoea and vomiting.*—This usually developed about eight days after an occlusion, which suggested a toxic symptom rather than an effect of the occlusion. Stools in all cases were negative for significant pathogens, but careful fluid and electrolyte control reduced the seriousness of this complication.

*Blepharitis and conjunctivitis* were noted in eight patients and in two progressed to corneal ulceration. The development of ocular symptoms was coincident with oral symptoms and the severity of this form of toxicity was directly related to the latter, as was the degree of *facial cutaneous hyperkeratosis.* Local treatment produced no significant response, but no case developed a residual corneal opacity.

*Alopecia and madarosis* were severe in all cases, but regrowth of the hair commenced three to four months after treatment had finished.

*Neurotoxicity.*—None of the fifteen patients treated developed symptoms of neurotoxicity.

*Periods of occlusion*

As will be noted from Table I it became apparent early in this investigation that a ten minute period of occlusion was not sufficient to protect the pelvic marrow depots. This has been fully discussed in the section on haematological toxicity. It was originally thought that the half life of dimethyl Myleran was shorter than that of nitrogen mustard (HN2) and that a ten minute period of occlusion would be adequate, but subsequent determinations by W. Davis (1962, personal communication) have given results of the order of twenty minutes. Of additional significance is the fact that dimethyl Myleran is a $S_N1$ type reactor and as such has a slower rate of fixation than the $S_N2$ type reactors such as HN2. The consequences of these differences are not confined to haematological toxicity as related to varying periods of occlusion ; the effective tumour dose is lowered if the circulating drug above the level of occlusion has not been fixed when the occluding tourniquet is released. This is well demonstrated in Cases 2 and 12. Case 2 received two courses each of 2·5 mg./kg. dimethyl Myleran. For the first course the periods of occlusion were ten minutes and only moderate tumour response was noted, whereas the occlusions for the second course were thirty minutes and a very marked regression ensued. The pattern of response was similar in Case 12. Eight courses of therapy using occlusion periods of ten minutes were completed, and each produced severe haematological toxicity. Three courses using a twenty minute period of occlusion produced severe haematological toxicity. Increasing the occlusion period to thirty minutes in four cases did not diminish the incidence of haematological toxicity. Consequently it was decided that a significantly longer period of occlusion was necessary. The possible complication of prolonged intestinal ischaemia (Marston, 1962) suggested that the intestinal tract should be sterilized before applying an occlusion for a sixty minute period. Twelve occlusions, each lasting for sixty minutes, have been used. In the first two courses sterilization of the intestinal tract was attempted using Chlorostrep ; subsequently this was not considered necessary. No neurological or other side effects attributable to the prolonged period of occlusion were noted. Haematological toxicity was diminished after the one hour occlusion periods and is discussed above.

*Tumour response in different malignancies*

*Anaplastic carcinoma of the post nasal space.*—Twelve cases with this disease (1–4, 6, 8–14) were treated with dimethyl Myleran and abdominal aortic occlusion. Four cases (1–4) had received previous therapy with HN2 administered as 1·0 mg./kg. intravenously over three days with minimal toxicity and slight to moderate tumour regression. Subsequent therapy with dimethyl Myleran administered as 2·5–3·5 mg./kg. with abdominal aortic occlusion produced marked haematological and buccal toxicity, with moderate tumour regression.

One case (12) had received two previous courses of therapy, i.e. 2·5 mg./kg. HN2 with A.A.O. and 1·6 mg./kg. chloramine mustard with A.A.O., each of which produced marked tumour regression. Treatment with 2·0 mg./kg. diethyl Myleran with ten minute occlusions produced much toxicity but no regression. A subsequent course of 2·4 mg./kg. with thirty minute occlusion produced marked tumour regression but fatal haematological toxicity. Tumour was not evident on post mortem examination.

Twelve patients with anaplastic carcinoma of the post nasal space were treated, in seven the response was classified as marked (Fig. 10 and 11), in three as moderate, in one as slight, and one case showed no response (*vide* Table I).

In no instance was the clinical response to dimethyl Myleran superior to that attained with comparable doses of nitrogen mustard (Clifford *et al.*, 1963), and of the twelve patients treated only one was discharged histologically free of disease (Case 10).

*Reticulum cell sarcoma.*—One case was treated (Case 5). Previous treatment with 1·0 mg./kg. HN2 had produced slight regression. Treatment with dimethyl Myleran, 2·5 mg./kg. and occlusion for ten minutes produced no anti-tumour effect. Subsequent therapy with oral Cytoxan was also ineffective.

*Solitary occipital plasmacytoma.*—One patient (Case 7) was treated and has been discussed. An almost identical case of occipital plasmacytoma had complete regression with nitrogen mustard 2·5 mg./kg. and A.A.O.

### *Conclusions*

Dimethyl Myleran administered with abdominal aortic occlusion requires an occlusion period of at least sixty minutes for complete drug fixation. The abdominal aorta can, with safety, be occluded for sixty minutes.

Dimethyl Myleran administered in the dosages, and with the technique, described is free from neurological toxicity, but bucco-labial mucositis and ulceration, blepharitis and conjunctivitis, thrombocytopenia, madarosis and alopecia are common, and may be serious complications with this form of therapy.

The upper safe limit of dosage for dimethyl Myleran administered intravenously without marrow protection is less than 2·0 mg./kg.

Eight out of the fifteen cases treated had marked tumour regression, and one was discharged from hospital histologically free of disease.

### SUMMARY

1. Fifteen cases of advanced malignant disease were treated with high doses of dimethyl Myleran and abdominal aortic occlusion.

2. The complications and results of this form of therapy are described, and the toxicity of dimethyl Myleran discussed.

We wish to thank Dr. F. L. Horsfall, Jr., and Dr. J. H. Burchenal of the Sloan-Kettering Institute for Cancer Research, who have provided a grant towards this work.

We also wish to thank Professor Alexander Haddow, F.R.S. and Dr. D. A. G. Galton for their encouragement, advice, and a supply of dimethyl Myleran.

We are indebted to Dr. C. A. Linsell, Dr. W. de C. Baker and Dr. S. H. Mehta, Medical Research Laboratory, Nairobi, for the histological studies, and to Dr. L. R. Whittaker for the radiological investigations undertaken on these patients.

We also wish to thank the other members of the Head and Neck Unit, King George VI Hospital, for their assistance in the medical and nursing care of these patients, and very special thanks to Mrs. Bradwell for her care and patience in the preparation of the manuscript.

### REFERENCES

Bierman, H. R., Kelly, K. H., Knudson, A. G. Jr., Maekawa, T. and Timmis, G. M.—(1958) *Ann. N.Y. Acad. Sci.*, **68**, 1211.
Clifford, P.—(1961) *J. Laryng.*, **75**, 707.
*Idem*, Clift, R. A. and Duff, J. K.—(1961) *Lancet*, i, 687.
*Idem*, Oettgen, H. F., Beecher, J. L., Brown, F. P., Harris, J. R. and Lawes, W. E.—(1963) *Brit. med. J.*, i, 1256.
Dameshek, W., Granville, N. B. and Rubio, F. Jr.—(1958) *Ann. N.Y. Acad. Sci.*, **68**, 1001.
Duff, J. K., Dennis, J., Clift, R. A., Clifford, P. and Oettgen, H. F.—(1961) *Brit. med. J.* ii, 1523.
Elson, L. A.—(1958) *Ann. N.Y. Acad. Sci.*, **68**, 826.
Haddow, A. and Timmis, G. M.—(1953) *Lancet*, i, 207.
Harries, J. R., Brown, F. P., Beecher, J. L. and Oettgen, H. F.—(1963) *Brit. med. J.* (In Press).
Hunt, C. C. and Phillips, F. S.—(1949) *J. Pharmacol.*, **95**, 131.
Marston, A.—(1962) *Lancet*, ii, 365.
Miller, D. G. and Lawrence, W.—(1961) *Proc. Amer. Ass. Cancer Res.*, **3**, 251.
Oettgen, H. F., Clifford, P., Beecher, J. L., Gillmore, J. H. and Clift, R. A.—(1964) *Klin. Wschr.*, **42**, 218.
Timmis, G. M. and Hudson, R. F.—(1958) *Ann. N.Y. Acad. Sci.*, **68**, 727.